# Commentary: Contextualising Maximal Fat Oxidation During Exercise: Determinants and Normative Values

**DOI:** 10.3389/fphys.2018.01460

**Published:** 2018-10-18

**Authors:** Francisco J. Amaro-Gahete, Guillermo Sanchez-Delgado, Jonatan R. Ruiz

**Affiliations:** ^1^Departament of Medical Physiology, School of Medicine, University of Granada, Granada, Spain; ^2^PROmoting FITness and Health Through Physical Activity Research Group, Department of Physical Education and Sports, Faculty of Sport Sciences, University of Granada, Granada, Spain

**Keywords:** MFO, FATmax, substrate oxidation, exercise, peak fat oxidation

We read with interest the study by Maunder et al. (2018) where they elegantly synthesized the available evidence regarding the biological factors that affect maximal fat oxidation (MFO) and the exercise intensity at which MFO occurs (Fatmax) (Maunder et al., 2018). Moreover, they compiled data from previous studies and provided normative values for MFO and Fatmax during exercise. Although we appreciate the usefulness of this approach, there are several important aspects that need to be considered.

Firstly, as Maunder et al. recognized, they provide percentiles for MFO and Fatmax derived from calculations based on mean and standard deviation rather than in true percentiles. This approach assumes a normal distribution of data, which may not be the case in studies with relatively small sample size.

Secondly, due to the lack of definitions of physical activity or fitness level in overweight and obese populations, Maunder et al. provided normative values for sedentary and physically active overweight/obese individuals without considering this important aspect. Several studies showed significant changes on MFO after an exercise intervention in overweight-obese individuals (Besnier et al., 2015; Rosenkilde et al., 2015). Therefore, the MFO and Fatmax normative values for the overweight and obese group should be considered with caution.

Thirdly, they compiled data from studies performed in cycloergometer. The mode of exercise (cycling, running, or walking) significantly influences MFO and Fatmax in young healthy and relatively fit individuals (Mendelson et al., 2012). However, its influence on sedentary people is unknown. Thus, it remains to be elucidated whether the provided normative values for MFO and Fatmax apply to the treadmill test.

Finally, Maunder et al. did not consider the potential effect of age on MFO and Fatmax, and, therefore, it was not taken into account in the normative values reported. Data from our laboratory (Table 1) suggest that age influences MFO, and, therefore, participants' age should be considered when providing normative values.

**Table 1:** Normative percentile values for maximal fat oxidation (MFO) and the exercise intensity at which maximal fat oxidation occurs (Fatmax) in sedentary individuals.

**Grouping criteria**	**Population**	**N**	**MFO (g/min)**	**20th percentile**	**40th percentile**	**60th percentile**	**80th percentile**	**Fatmax (%VO_2_max)**	**20th percentile**	**40th percentile**	**60th percentile**	**80th percentile**
All	Sedentary adults	167	0.34 ± 0.10	0.24	0.30	0.35	0.42	44.2 ± 12.4	33.2	39.6	44.6	54.1
By Sex	Sedentary adult men	60	0.37 ± 0.11	0.29	0.34	0.38	0.44	40.8 ± 11.0	32.2	37.2	41.1	48.8
	Sedentary adult women	107	0.32 ± 0.10	0.24	0.28	0.33	0.40	46.1 ± 12.8	34.8	42.1	47.8	55.9
By Age	Sedentary young adults	125	0.36 ± 0.11	0.28	0.32	0.36	0.44	44.0 ± 13.3	32.5	39.0	43.2	54.6
	Sedentary middle-aged adults	42	0.29 ± 0.08	0.22	0.24	0.28	0.38	44.7 ± 9.5	35.8	41.4	46.0	53.4
By Sex and Age	Sedentary young adult men	41	0.38 ± 0.12	0.28	0.35	0.38	0.48	39.8 ± 11.7	29.4	36.7	40.3	48.6
	Sedentary young adult women	84	0.35 ± 0.09	0.28	0.31	0.35	0.42	46.1 ± 13.6	34.4	41.6	46.6	60.4
	Sedentary middle-aged adult men	19	0.35 ± 0.07	0.29	0.33	0.38	0.42	43.0 ± 9.3	35.5	39.5	44.4	53.1
	Sedentary middle-aged adult women	23	0.24 ± 0.03	0.22	0.23	0.24	0.27	46.1 ± 9.6	39.0	42.7	50.1	54.4
By BMI Status	Normal-weight sedentary adults	88	0.34 ± 0.11	0.25	0.30	0.34	0.42	45.3 ± 12.7	35.1	41.1	46.5	55.6
	Overweight sedentary adults	50	0.33 ± 0.09	0.24	0.30	0.35	0.41	42.7 ± 11.2	32.7	39.1	42.7	53.1
	Obese sedentary adults	29	0.36 ± 0.12	0.26	0.30	0.39	0.45	43.3 ± 13.5	32.3	39.1	42.0	54.8

*Data are presented as mean (standard deviation). BMI, Body mass index; min, Minute; VO_2_max, Maximum oxygen uptake*.

Here, we provide normative values by sex, weight status, and age for MFO and Fatmax (Table 1) of 167 (*n* = 107 women) sedentary healthy individuals evaluated by a treadmill test. We determined the MFO and Fatmax in 125 young adults aged 22.1 ± 2.2 years old [84 women, body mass index (BMI): 25.0 ± 4.8 kg/m^2^] (Sanchez-Delgado et al., 2015) and in 42 middle-aged adults aged 52.1 ± 4.6 years old [23 women, BMI: 27.8±3.6 kg/m^2^] (Amaro-Gahete et al., 2018). We conducted a graded exercise protocol on a treadmill that started with a 3-min warm-up at 3.5 km/h (gradient 0%) and continued with speed increments of 1 km/h every 3 min until the maximal walking speed was reached. The treadmill speed was kept constant with the gradient increasing by 2% every 3 min until the respiratory exchange ratio was ≥1.0 (Jeukendrup and Wallis, 2005). Fat oxidation was calculated during the last 60 s of each step using a stoichiometric equation for respiratory gas exchange (Frayn, 1983) disregarding protein oxidation. A third polynomial curve with intersection at 0;0 (Croci et al., 2014) was determined for each individual in order to determine MFO and Fatmax.

Our results showed that absolute MFO was higher in men than in women (0.37 ± 0.11 vs. 0.32 ± 0.10 g/min, respectively, *P* = 0.004, see Table 1), while Fatmax was lower in men than in women (40.8 ± 10.99 vs. 46.1 ± 12.84% VO2max [maximum oxygen uptake], respectively, *P* = 0.009, see Table 1). Considering the known sex-related differences in body composition, MFO relative to fat free mass (FFM) might be more appropriate when conducting sex comparisons (Venables et al., 2005; Fletcher et al., 2017; Maunder et al., 2018). Our results showed that MFO relative to FFM (assessed by dual-energy X-ray absorptiometry) was lower in men than in women (0.050 ± 0.026 vs. 0.084 ± 0.043 g/min/kg, respectively, *P* < 0.001). These findings concur with those presented by Maunder et al. (2018), who showed that absolute MFO was greater in physically active men than in women (0.56 vs. 0.33 g/min, respectively), whereas Fatmax was slightly higher in physically active women than in men (56.0 vs. 51.0% VO2max, respectively).

A recent study described the MFO and Fatmax values in an athletic population across different ages, and showed large inter-individual differences regardless of the sport modality (Randell et al., 2017). Our results showed significantly higher MFO in young compared with middle-aged sedentary adults (0.36 ± 0.11 vs. 0.29 ± 0.78 g/min, respectively, *P* < 0.001), whereas no differences were observed in Fatmax (44.0 ± 13.30 vs. 44.7 ± 9.47 % VO2max, respectively, *P* = 0.753).

Furthermore, we reported MFO and Fatmax normative values by weight status in sedentary adults. We observed similar MFO and Fatmax values in normal-weight, overweight, and obese individuals (MFO: 0.34 ± 0.11, 0.33 ± 0.09, and 0.36 ± 0.12 g/min, respectively, *P* = 0.494; Fatmax: 45.9 ± 12.9 vs. 42.6 ± 10.9 vs. 43.3 ± 13.5% VO2max, respectively, *P* = 0.146). In contrast, Maunder et al. (2018) showed lower MFO in obese individuals, which may be due to differences in training status, since Maunder et al. (2018) did not consider this dimension in the obese population.

It should be noted that the cohorts included in Maunder et al. review performed a graded exercise protocol test after an overnight fast, whereas the participants in our study fasted only for 5–6 h. Previous studies suggested that the nutritional status plays an important role in MFO and Fatmax determination (Achten and Jeukendrup, 2003; Fletcher et al., 2017; Amaro-Gahete and Ruiz, 2018; Maunder et al., 2018; Purdom et al., 2018), and, therefore, fasting should be carefully considered when determining MFO and Fatmax.

We believe that the normative values provided here and those by Maunder et al. will be very useful when evaluating MFO and Fatmax both in research and in clinical settings. However, whenever possible, future studies should provide normative data by sex, age, training status, and weight status.

## Data availability statement

The raw data supporting the conclusions of this manuscript will be made available by the authors, without undue reservation, to any qualified researcher.

## Author contributions

FA-G, GS-D, and JR-R fully reviewed and criticized the original article, drafted the commentary, reviewed, and approved the final manuscript.

### Conflict of interest statement

The authors declare that the research was conducted in the absence of any commercial or financial relationships that could be construed as a potential conflict of interest.
